# Post-Partum Thrombotic Thrombocytopenic Purpura (TTP) in a Patient with known Idiopathic (Immune) Thrombocytopenic Purpura: a case report and review of the literature

**DOI:** 10.1186/s13256-018-1692-1

**Published:** 2018-06-01

**Authors:** Naser Al-Husban, Oqba Al-Kuran

**Affiliations:** Faculty of Medicine, University of Jordan and Jordan University Hospital, PO Box 2194, Amman, 11941 Jordan

**Keywords:** ITP, TTP, Postpartum, Plasmapheresis

## Abstract

**Background:**

Incidences of immune thrombocytopenic purpura occur in 1 in every 1000–10,000 pregnancies accounting for 3% of all thrombocytopenic pregnancies. A pre-existing immune thrombocytopenic purpura is known to be a risk factor for developing thrombocytopenia during pregnancy. We present here the treatment regime and management of a patient with known immune thrombocytopenic purpura who developed postpartum thrombotic thrombocytopenia with atypical response to traditional therapy.

Pregnant women are more vulnerable to immune thrombocytopenic purpura or thrombotic thrombocytopenia. Pregnancy or postpartum thrombotic thrombocytopenia accounts for 10–25% of all thrombotic thrombocytopenia.

**Case presentation:**

This case report deals with the treatment regime and management of a patient with known immune thrombocytopenic purpura who developed postpartum thrombotic thrombocytopenia. A 30-year-old Middle Eastern woman, with a prior diagnosis of chronic immune thrombocytopenic purpura had remained off-the-treatment for many years. After primary unexplained infertility for 8 years, for which she underwent six failed trials of *in vitro* fertilization, she delivered a healthy baby through caesarean section. Two days post-surgery, she had persistent thrombocytopenia, ecchymoses, bruises, and hemolysis. Her blood film revealed leukoerythroblastic anemia. Her blood tests also revealed a very low level of haptoglobin, and low level of ADAMTS13. A diagnosis of thrombotic thrombocytopenia was suspected. Plasma exchange therapy was started with partial response. We showed that rituximab in conjunction with mycophenolate mofetil following plasma exchange therapy was effective in controlling the low platelet count in our patient.

**Conclusions:**

Rituximab in conjunction with mycophenolate mofetil following plasma exchange therapy was effective in controlling the low platelet count in our patient. Only two doses of rituximab were sufficient to normalize our patient. We present here a case of safe and effective use of rituximab in pregnancy-induced thrombotic thrombocytopenia.

## Background

The occurrence of immune thrombocytopenic purpura (ITP) or thrombotic thrombocytopenia (TTP) is greatly increased during pregnancy. Incidences of ITP occur in 1 in every 1000–10,000 pregnancies accounting for 3% of all thrombocytopenic pregnancies [1]. Pregnancy or postpartum-related TTP accounts for 10–25% of all TTP. A pre-existing ITP is known to be a risk factor for developing thrombocytopenia during pregnancy [2]. We present here the treatment regime and management of a patient with known ITP who developed postpartum TTP with atypical response to traditional therapy. The unusualness of this case was the onset of presentation (postpartum) and the bruises and ecchymosis which were the main clinical features. These features are not known to be associated with ITP and medical staff has to be very vigilant and careful with these cases.

## Case presentation

A 30-year-old married Middle Eastern woman, a housewife living in a big city in Jordan, with a prior diagnosis of chronic ITP remained off-the-treatment for many years. She was not known to have any other medical illness. There was no family history of ITP or TTP. She had never smoked tobacco, drunk alcohol, or used illicit drugs. She was investigated for other causes of thrombocytopenia including systemic lupus erythematosus (SLE)-antiphospholipid antibody syndrome (APS) but, unfortunately, the laboratory-specific results were not available, however, our patient stated that they were negative. Initially she was prescribed a low dose of orally administered prednisolone but this was discontinued years ago. She remained asymptomatic with approximate whole blood platelet counts of 50 × 10^9^/L. She had primary unexplained infertility for 8 years for which she underwent six failed trials of *in vitro* fertilization (IVF). She conceived spontaneously and remained asymptomatic with whole blood platelet counts > 50 × 10^9^/L. At 26 weeks of gestation, she was diagnosed as having glucose intolerance and started on metformin 850 mg twice daily. At 30 weeks of gestation, her whole blood platelet count was 30 × 10^9^/L and she was still asymptomatic. She was started on 15 mg orally administered prednisolone. One week later, her whole blood platelet count dropped to 28 × 10^9^/L, her prednisolone dose was increased to 30 mg daily. She was screened for lupus anticoagulant, anticardiolipin antibody (ACA), and anti-beta-2 glycoprotein 1 (BTIIGLYI) antibody and the results were normal: partial thromboplastin time (PTT) 27 seconds, ACA-immunoglobulin G (IgG) < 14 GPL, ACA-IgM < 12 MPL, BTIIGLYI-IgM 16 U/mL, and BTIIGLYI-IgG 17 U/mL. Two weeks later, her whole blood platelet count was 56 × 10^9^/L. Her prednisolone dose was tapered gradually down to 15 mg at 34 weeks of gestation. At 35 weeks, her whole blood platelet count was 45 × 10^9^/L, her blood hemoglobin (Hb) level was 125 g/L with an A-positive blood group, and she was asymptomatic with normal blood pressure (BP) and urine analysis.

By 36 weeks of gestation, she presented with ruptured membranes, with no uterine contractions. Her BP, oral temperature, and pulse rate were 120/70 mmHg, 37.1 degrees Celsius and 90 beats/minute, respectively. Her general examination was unremarkable. An obstetric examination revealed a cephalic presentation and a fundal height that was corresponding to a 36-week pregnancy. There were no skin lesions. Her neurological examination was normal with no focal neurological deficits. A cardiotocography (CTG) was performed and was normal. Her Hb, white blood cell (WBC) count, and platelet count were 130 g/L, 12 × 10^9^/L, and 33 × 10^9^/L, respectively. Her liver function tests were normal: lactate dehydrogenase (LDH) 3.0 ukat/L and bilirubin total 8 umol/L. She insisted on caesarean section delivery despite thorough counselling regarding the mode of delivery. She was given intravenously 500 mg of methylprednisolone and intramuscularly 1500 IU of anti-D immunoglobulin in an attempt to quickly raise her platelet count. Six hours later, an uncomplicated caesarean section was performed under spinal anesthesia (she refused to have a general anesthetic and requested a spinal anesthesia despite counselling regarding the risk of hematoma because of her low platelet count). A healthy baby was delivered weighing 2.3 kg with normal platelet count. No instance of bleeding was noted during the surgery. The operative blood loss was estimated to be around 1000 ml. Recovery after surgery was smooth. She was given low molecular weight heparin (LMWH) (40 mg of enoxaparin sodium), subcutaneously 12 hours after operation.

Postoperatively on day 1, she was doing well with stable vital signs and no abnormal vaginal bleeding. No incidence of bruises or ecchymosis around the wound or anywhere else was noted. Her platelet count, WBC, and Hb were 46 × 10^9^/L, 18.35 × 10^9^/L, and 96 g/L, respectively. She was started on 30 mg of orally administered prednisolone. On the second postoperative day, ecchymosis started to appear around the wound. Her BP and urine analysis were normal. She had a platelet count of 52 × 10^9^/L. Although she was reassured about the safety of breast feeding, she was not happy to breast feed and she had no breast pain or engorgement. In the afternoon, the bruises and ecchymosis spread across her lower abdomen and down to her upper thighs. A hematologist was consulted who advised observation only.

On the third postoperative day, the ecchymosis expanded bilaterally over her suprapubic area, vulval area, flanks, and thighs. She complained of occipital headache which was relieved by simple analgesia, there were no other neurological symptoms or signs. Her BP was normal. An abdominal ultrasound (U/S) scan revealed an empty uterus with no free fluid in her abdomen or pelvis and no evidence of presence of hematoma. Her whole blood Hb was 88 g/L, WBC was 12.89 × 10^9^/L, and platelet count was 50 × 10^9^/L. We suspected a hemolytic process so a liver function test, haptoglobin, and blood film were requested. These revealed total serum bilirubin 17.07 umol/L, direct serum bilirubin 5.97 umol/L, total serum protein 55.6 g/L, serum albumin 34.4 g/L, serum alanine aminotransferase (ALT) 0.47 ukat/L, serum aspartate aminotransferase (AST) 0.80 ukat/L, serum gamma glutamyltransferase (GGT) 0.55 ukat/L, and serum LDH 21.1 ukat/L. Her serum haptoglobin level was low, 100 mg/L, with negative direct and indirect Coombs tests. Her urine was normal. Blood film showed normocytic red cell anemia, polychromasia, few schistocytes, one to two nucleated red blood cells (RBCs)/100 WBCs, neutrophilic leukocytosis, occasionally left shifted neutrophils, and thrombocytopenia with large forms. Three ampoules of iron sucrose complex were given intravenously over 3 hours and she was started on orally administered iron.

Four days after surgery, she was doing well apart from recurrent attacks of headache. Her BP was normal and she had no focal neurological signs. Her serum vitamin B12 level, serum iron, serum folate level, serum ferritin level, plasma prothrombin time (PT), whole blood PTT, and plasma fibrinogen were normal. Her newborn’s platelet count was normal. Total as well as direct bilirubin and LDH were elevated: 26.4 umol/L, 7.2 umol/L, and 26.7 ukat/L, respectively. Five days after surgery, there were severe and extensive bruises as well as ecchymosis. No abnormal vaginal bleeding or epistaxis was seen. Orally administered mycophenolate mofetil 360 mg was started twice daily in addition to the daily 30 mg orally administered prednisolone she was already taking.

On the sixth postoperative day, ecchymosis was seen all over her abdomen, flanks, thighs, legs, and low back area. Her Hb was 83 g/L, WBC 16.6 × 10^9^/L, platelet count 50 × 10^9^/L, and LDH 28.7 ukat/L, and she had normal total bilirubin, direct bilirubin, random serum glucose, sodium, potassium, urea, and creatinine. Urine analysis showed + 1 proteinuria, no sugar, one to two WBC/high power field (HPF), and 2–4 RBC/HPF. Indirect and direct Coombs tests were negative. Two ampoules of iron sucrose were administered intravenously in 2 hours. Prednisolone dose was increased to 1 mg/kg daily.

Bruises and ecchymosis increased 1-week post-surgery. Blood film revealed leukoerythroblastic anemia, anisocytosis, nine nucleated red cells/100 WBC, 8% left shifted neutrophils, and thrombocytopenia with few large forms. Her LDH level was 28.4 ukat/L. Her platelet count was 56 × 10^9^/L; total bilirubin, direct bilirubin, ALT, AST, and urine analysis were normal. TTP was suspected; hence, ADAMTS13 test was requested. The result indicated 11%. The next day, her bruises and ecchymosis were extensive and spreading. She was doing well with no neurological symptoms and stable vital signs. Her urine was normal. Blood film revealed plenty of schistocytes. TTP was strongly suspected; she was given 5 units of fresh frozen plasma (FFP). Eight hours after the FFP infusion, her platelet count was 76 × 10^9^/L.

Bruises decreased the day after FFP infusion. Plasmapheresis (a left femoral dialysis line was inserted under aseptic technique) was started. The first session was done with 10 units of FFP and 4 units of cryoprecipitate. Investigations showed normal Hb, WBC, platelet count, liver function tests, and urine. On the successive day, bruises and ecchymosis decreased. LDH was 15.8 ukat/L with normal creatinine, calcium, phosphorous, magnesium, total bilirubin, direct bilirubin, ALT, AST, GGT, PT, PTT, and fibrinogen level. A urine examination showed + 1 proteinuria. A second session of plasmapheresis was done. On the next day, her platelet count was 60 × 10^9^/L and Hb was 99 g/L with normal liver and kidney functions. Her LDH was 13.7 ukat/L, with normal PT, PTT, and fibrinogen level. Blood film showed leukoerythroblastic anemia, anisocytosis, polychromasia, schistocytes, and spherocytes. A third session of plasmapheresis was done. Following FFP infusion and plasmapheresis, ADAMTS13 level was > 19%.

On the 12th postoperative day, a fourth session of plasmapheresis was done. Her LDH was 10.8 ukat/L with normal kidney and liver functions. Her platelet count was 54 × 10^9^/L.

After the fifth session of plasmapheresis, LDH was 8.56 ukat/L. PT, PTT, and fibrinogen level were normal. Ecchymosis decreased substantially. Our patient was asking for discharge.

The next day, her platelet count was 52 × 10^9^/L; LDH was 13.4 ukat/L with normal liver and kidney functions. A sixth session of plasmapheresis was carried out. A urine examination showed + 1 proteinuria. During all these days, our patient was on 1 mg/kg orally administered prednisolone per day and 360 mg orally administered mycophenolate twice daily. Ecchymosis decreased further on the following day. She was given intravenously rituximab 500 mg in 5 hours. Her LDH level was 11.0 ukat/L, platelet count was 100 × 10^9^/L, Hb 111 g/L, and WBC 7.92 × 10^9^/L. She was discharged with a prescription for orally administered prednisolone 1 mg/kg daily and 360 mg of orally administered mycophenolate twice daily.

One week later, she was given the second dose of rituximab as an out-patient. She continued to do well with no bleeding tendency and a platelet count swinging between 50 × 10^9^/L and 60 × 10^9^/L.

At 4 weeks after surgery, her Hb, WBC, and platelet count were 128 g/L, 13.7 × 10^9^/L, and 130 × 10^9^/L, respectively. Her liver and kidney function tests were normal. Two weeks later, her platelet count was 80 × 10^9^/L. After a further 2 weeks, she continued to receive the same mycophenolate dose but started to decrease her prednisolone dose until it was completely stopped 3 weeks later.

Ten weeks after surgery, she became asymptomatic with a platelet count of 92 × 10^9^/L. She was receiving only mycophenolate mofetil at a dose of 360 mg twice daily. She stopped mycophenolate mofetil and remained completely asymptomatic. Six months after delivery, she was asymptomatic with a platelet count of 85 × 10^9^/L. One year after her delivery, she was doing well, asymptomatic, and her platelet count was 79 × 10^9^/L. Table 1 refers to results of patient’s blood tests in a chronological order.

**Table 1:** Shows results of patient’s blood tests in a chronological order

Days	1	2	3	4	5	6	7	8	9	10	11	12	13	14
Hb (g/L)	96		88			83					99			111
Platelets (×10^9^/L)	46	52	50			50	49				60	54	52	100
WBC (×10^9^/L)	18.35		12.89			16.6								7.9
Total bilirubin (umol/L)			17.07	26.4										
Direct bilirubin (umol/L)			5.97	7.2										
Total protein (g/L)			55.6											
Albumin (g/L)			34.4											
ALT (ukat/L)			0.47											
AST (ukat/L)			0.80											
GGT (ukat/L)			0.55											
LDH (ukat/L)			21.1	26.7		28.7	28.4			15.8	13.7	10.8	13.4	11.0
Haptoglobin (mg/L)			100											
Direct Coombs			Negative				Negative							
Indirect Coombs			Negative				Negative							
Serum glucose (mmol/L)						4.1								
Serum creatinine (umol/L)						90								

*ALT* alanine aminotransferase, *AST* aspartate aminotransferase, *GGT* gamma glutamyltransferase, *Hb* hemoglobin, *LDH* lactate dehydrogenase, *WBC* white blood cells

## Discussion

This was a case of pregnancy-triggered TTP in a patient with chronic ITP. Moreover, she underwent a caesarean section under a spinal anesthetic with severe thrombocytopenia and did not experience hemorrhagic complications. It unusually presented in the immediate postpartum period and was not a typical TTP; it did not respond to the traditional management pathways.

TTP is a rare life-threatening disease which requires rapid intervention. Pregnancy is a known trigger for thrombocytopenia. A previous history of ITP aggravates thrombocytopenia in pregnancy [2]. However, to date it is diagnosed by exclusion of other diseases due to lack of concrete biomarkers [3]. In this case report we present a case in which a chronic history of ITP which remained asymptomatic for years was precipitated as TTP after delivery. Further, we discuss the difficulties encountered in the diagnosis of TTP in the postpartum woman as well as the management of the patient.

A corticosteroid, such as prednisolone, is the first line of therapy for ITP [4]. It increases the platelet count in 2–4 weeks. However, in our case, the intake of a high dose of orally administered prednisolone initially with doses tapering in the later part making a total of 4 weeks was ineffective in increasing our patient’s platelet count. Intravenous administration of anti-D with or without steroids increases the platelet count rapidly in patients with ITP [5]. It is suitable for Rhesus antigen D (RhD) positive patients who are not splenectomized as in our case. Baseline Hb has a significant impact on platelet response to anti-D in adults [5]. Anti-D is also known to cause hemolysis and this is more pronounced 1 day after its administration [5]; this was not the scenario in our case. Similarly, methylprednisolone is more effective than prednisolone in increasing the platelets count in thrombocytopenia [6]. Administration of methylprednisolone and anti-D a few hours before delivery by caesarean section in our patient was effective in preventing excessive blood loss.

However, except for thrombocytopenia and microangiopathic hemolytic anemia, which occur frequently (50–75%), other symptoms of TTP are either not present or vaguely observed. Therefore, thrombocytopenia, microangiopathic hemolytic anemia, and absence of alternative etiology are sufficient to diagnose TTP [6, 7]. This allows physicians to diagnose TTP rapidly, which can be of lifesaving importance. Incidences of TTP are also increasingly associated with pregnancy [8]. TTP can commence at any time in pregnancy. In a study, out of 13 pregnancies complicated by thrombotic microangiopathies (TMA), three patients had onset before mid-pregnancy, eight had onset during peripartum, and two several weeks postpartum [9]. In our case, thrombocytopenia was detected during the third trimester while TTP was symptomatologically evident (by ecchymosis) at second day after delivering the baby by caesarean section. In our case, even though thrombocytopenia was present during the third trimester of gestation, the hemolytic anemia precipitated on second postoperative day. With the exception of the recurrent attacks of headache and proteinuria, her renal function and nervous system were normal without any episodes of fever. Because TTP typically does not have a bleeding tendency, the possibility of a migrating subcutaneous hematoma after the caesarean section with spinal anesthesia, which could have been facilitated by the single dose of LMWH, was excluded by a normal U/S scan of her abdominal wall, abdominal cavity, and pelvis.

Severe deficiency of ADAMTS13 is the underlying cause for developing TTP [3]. In acquired TTP this is mediated by auto-antibodies against ADAMTS13. An ADAMTS13 value of < 10% is indicative of TTP. However, ADAMTS13 levels can also be lowered (but > 10%) in pregnancy, postpartum, and other non-TTP diseases such as hemolytic uremic syndrome, hematopoietic stem cell and solid organ transplantation, liver disease, disseminated intravascular coagulation (DIC), sepsis, certain medications [3], preeclampsia, eclampsia, and hemolysis, elevated liver enzymes, and low platelet count (HELLP) [10, 11]. Moreover, interferences in the assay measuring ADAMTS13 can occur from a high concentration of von Willebrand factor (vWF), hyperlipidemia, hemolysis with plasma free Hb > 2 g/L, and hyperbilirubinemia, and cleavage by other proteases can even complicate identification of TTP [3]. Besides, recent plasma exchange or transfusion may falsely normalize ADAMTS13 levels, thus potentially masking the diagnosis of TTP. Therefore, TTP remains a challenge in clinical diagnosis. In our patient, ADAMTS13 activity was 11%. Although undetectable levels of the enzyme activity are diagnostic of inherited or acquired TTP, not all patients diagnosed as having TTP have severe protease deficiency, and it is therefore not recommended as an initial test for diagnosis. Therefore, based only on the ADAMTS13 level, a concrete diagnosis of TTP is difficult. The rise in the level of ADAMTS13 activity with plasma infusion and plasmapheresis was in support of a diagnosis of TTP. Moreover, support from other laboratory diagnoses is very necessary.

Identifying the presence of hemolytic anemia and its underlying cause is of prime importance in diagnosing TTP. The presence of schistocytes, the fragmented RBCs injured by damaged endothelium, in blood film investigation is one of the strong indicators of hemolytic anemia [12, 13]. However, identifying it microscopically lacks standardization and is dependent on the evaluator. A threshold of 0.2–0.5% schistocytes is necessary before suspecting TTP. In our case the few schistocytes were observed on third day post-surgery which increased drastically in 5 days. Although hemolysis (fragmented RBCs) was indicative in our case the decrease in Hb in our patient was not drastic (Hb changed from 96 to 83 g/L in 1 week). However, schistocytes are not specific for TTP, but are also seen in healthy persons as well as in patients with preeclampsia, eclampsia, chronic renal failure, solid organ or bone marrow transplantation, diabetic microangiopathy, and SLE, as well as in patients with a prosthetic heart valve [14, 15]. Severe bleeding is a common cause for the appearance of nucleated RBCs in peripheral blood smears in addition to reticulocytosis, but our patient did not have any bleeding episode. Therefore, the laboratory findings which appeared important in the diagnosis of TTP were high LDH to AST ratio since an increased LDH-to-AST ratio is known to indicate TTP [16, 17]. High LDH levels along with elevated creatine kinase are observed after surgery including caesarean section and are not always conclusive for hemolysis. Usually a negative Coombs test shows that the reason for hemolysis is TTP while a positive Coombs test suggests an immune-mediated reason for hemolysis [18]. Other causes of secondary TTP like DIC or APS are unlikely in our case because our patient was screened negative for APS and she had no excessive bleeding or blood transfusion to indicate a possible DIC.

Early diagnosis of TTP and management of its symptoms acutely with advanced techniques such as plasma exchange therapy (PEX) is necessary as delay in initiating PEX is known to cause adverse outcomes [3, 19]. Normally, two to three sessions of PEX are sufficient to stabilize the platelet count [3], however, in our case, our patient achieved only symptomatological (ecchymosis decreased) relief and her LDH began to decrease after administration of PEX. Her platelet count failed to reach normal levels while her Hb level was also within 99 g/L range. Administration of immunosuppressant mycophenolate mofetil as well as corticosteroid prednisolone along with PEX was ineffective in increasing her platelet count and Hb level. In a few cases of ITP and TTP, mycophenolate mofetil has been shown to increase the platelet count as well as Hb [20–22].

Rituximab is known to be a safe and effective treatment for newly diagnosed TTP and ITP; it decreases the number of PEX required for achieving remission and lowers the risk for recurrence by approximately 80%. In addition, unresponsiveness to steroids in chronic ITP is relatively common and fast responses to rituximab can be seen. Diagnostic consideration, therefore, cannot depend on the response to these therapies since they are both used effectively in both ITP and TTP [23, 24]. However, a review of 231 pregnancies indicated that rituximab is able to cross the placental barrier and induce neonatal hematological abnormalities or malformations [25]. Contrarily, in a study by Scully *et al.*, administration of rituximab in a woman with pregnancy-related TTP was found to be safe and effective [26]. Application of rituximab along with PEX or shortly after PEX requires a greater number of applications of the medication to be effective because rituximab is removed from the circulation by the PEX [27]. Intravenous supply of rituximab, following PEX, not only rapidly increased the platelet count and Hb in our patient but also controlled her WBC count, which was slightly elevated, and normalized her LDH level. Our treatment regime of first performing multiple PEX to remove the inhibitors of ADAMTS13 and then applying rituximab was more effective and rapid. After a second dose of rituximab our patient was completely normal with normal platelet counts. PEX may have actually worked to correct the hemolysis but not the thrombocytopenia because she has an underlying ITP and ITP does not respond to PEX (this might explain why there was no prompt platelet increase whereas LDH levels dropped and Hb levels rose quickly). Rituximab may have worked both sided (ITP and TTP) and would explain the platelet rise.

## Conclusions

The ADAMTS13 level of our patient of 11% made the diagnosis of TTP difficult. Moreover, the PEX applications were ineffective in managing her platelet and Hb levels to normal. Therefore, the impact of ADAMTS13 levels and the presence of its inhibitors on overall survival, ultimate clinical outcome, responsiveness to plasma exchange, and relapse in pregnancy-related TTP are still controversial. Therefore, studies assessing this clinical correlation are recommended. We show that rituximab in conjunction with mycophenolate mofetil following PEX was effective in controlling the low platelet count in our patient. Only two doses of rituximab were enough to normalize our patient. We present here a case of safe and effective use of rituximab in pregnancy-induced TTP.

## Abbreviations

ACA: Anticardiolipin antibody
ALT: Alanine aminotransferase
APS: Antiphospholipid antibody syndrome
AST: Aspartate aminotransferase
BP: Blood pressure
BTIIGLYI: Beta-2 glycoprotein 1
CTG: Cardiotocography
DIC: Disseminated intravascular coagulation
FFP: Fresh frozen plasma
GGT: Gamma glutamyltransferase
Hb: Hemoglobin
HELLP: Hemolysis, elevated liver enzymes, and low platelet count
HPF: High power field
ITP: Immune thrombocytopenic purpura
IVF: *In vitro* fertilization
LDH: Lactate dehydrogenase
LMWH: Low molecular weight heparin
PEX: Plasma exchange therapy
PT: Prothrombin time
PTT: Partial thromboplastin time
RBC: Red blood cell
SLE: Systemic lupus erythematosus
TMA: Thrombotic microangiopathies
TTP: Thrombotic thrombocytopenia
U/S: Ultrasound
vWF: Von Willebrand factor
WBC: White blood cell
